# Viscocohesive hyaluronan gel enhances stability of intravital multiphoton imaging with subcellular resolution

**DOI:** 10.1117/1.NPh.12.S1.S14602

**Published:** 2024-11-22

**Authors:** Ryan A. Morton, Tyson N. Kim

**Affiliations:** a University of California San Francisco, Department of Ophthalmology, San Francisco, California, United States; b UCSF-UC Berkeley Graduate Group in Bioengineering, San Francisco/Berkeley, California, United States

**Keywords:** multiphoton microscopy, intravital imaging, hyaluronan gel, viscoelastic, viscocohesive, immersion media

## Abstract

Multiphoton microscopy (MPM) has become a preferred technique for intravital imaging deep in living tissues with subcellular detail, where resolution and working depths are typically optimized utilizing high numerical aperture, water-immersion objectives with long focusing distances. However, this approach requires the maintenance of water between the specimen and the objective lens, which can be challenging or impossible for many intravital preparations with complex tissues and spatial arrangements. We introduce the novel use of cohesive hyaluronan gel (HG) as an immersion medium that can be used in place of water within existing optical setups to enable multiphoton imaging with equivalent quality and far superior stability. We characterize and compare imaging performance, longevity, and feasibility of preparations in various configurations. This combination of HG with MPM is highly accessible and opens the doors to new intravital imaging applications.

## 1 Introduction

Multiphoton microscopy (MPM) has become the method of choice for imaging deep into tissues and organisms with subcellular resolution, providing outstanding detail of biological dynamics in vivo, often over extended time periods.^1^^–^^6^ MPM has been transformative in many research areas, including neurobiology, where it enables the visualization of intricate neurovascular structure, neural activity, and synaptic dynamics;^4^^,^^7^^–^^15^ immunology, offering insights into immune cell behaviors and interactions critical for healthy and pathologic immune responses;^16^^–^^18^ cancer biology, for studying tumorigenesis, metastasis, and immune interaction in disease and treatment;^19^^–^^22^ and developmental biology, aiding exploration of cell differentiation, lineage tracing, organ formation, developmental disorders, and tissue regeneration.^23^^–^^25^ The utility of MPM stems from its unique ability to generate localized signals deep within a specimen while minimizing photobleaching and damage to surrounding tissues. This is achieved through the use of tightly focused, femtosecond laser pulses to induce multiphoton excitation, a process wherein two or more photons simultaneously excite a molecule that would normally not be excited at a given wavelength through single photon interactions.^1^^,^^2^^,^^6^ This technique allows for the generation of fluorescence from endogenous tissues, exogenous probes, and genetically encoded fluorescent proteins, wherein multiphoton excitation facilitates the transition of a fluorophore from its ground to an excited state.^1^^,^^2^^,^^6^ In addition, MPM is sensitive to second and third harmonic generation in certain tissues with highly organized molecular arrangements,^26^^–^^29^ where two or three photons of light are converted into a single photon of equivalent energy and proportionally shorter wavelength.

Deep intravital MPM typically employs water-immersion objectives to achieve subcellular resolution at extended working depths. MPM requires tight focusing of laser light through a high numerical aperture (NA) objective to produce small excitation volumes necessary for high-resolution imaging;^30^^–^^32^ however, tighter focusing in a given media shortens the working distance. As such, water immersion has become important for intravital MPM as it can extend working depths while preserving the high NA required for multiphoton excitation with subcellular resolution. Consequently, intravital MPM assays often require sophisticated preparations or surgical procedures to expose target tissues while maintaining a stable presence of immersion water. For example, brain imaging often involves creating a cranial window by removing a portion of the skull and surrounding this window with a rim of dental cement, thereby forming a reservoir that can be filled with water for a water-immersion lens.^5^^,^^9^^,^^33^ Other complex surgical preparations have been established for multiphoton imaging of organs such as the eye,^34^^–^^36^ heart,^37^ gastrointestinal system,^38^ liver,^39^ kidney,^40^ and lymphoid tissue.^16^^,^^20^ Each preparation is uniquely designed for its target tissue yet shares the common goal of preserving a reservoir for water-immersion imaging over an extended period.

Water-immersion microscopy has inherent limitations due to water’s limited surface tension, the tendency to wick easily into fibers and hair, and difficulty with arranging some tissues in a fashion suitable for a stable water interface. For example, the curvature and small size of the rodent’s eye present significant challenges for water immersion over extended periods, particularly when repositioning the eye is necessary to image different regions in a single session.^34^^–^^36^ A novel approach to overcome these hurdles is inspired by modern surgical techniques in ophthalmology. Hyaluronan gels (HGs), also known as ophthalmic viscosurgical devices, are transparent and exceptionally cohesive liquids that are injected into the eye to stabilize and protect delicate intraocular structures, minimize fluid loss, and enhance visualization during surgery.^41^^–^^43^ Comprised mainly of hyaluronic acid, water, and sometimes chondroitin sulfate, HGs are formulated to offer exceptionally high viscosity and cohesive properties while maintaining a refractive index closely aligned with water to minimize or eliminate optical distortions during surgical visualization.^41^^,^^42^ The advantages of using HG for immersion microscopy are considerable. HG resists evaporation and wicking, can have remarkably high cohesion and surface tension, and is engineered to be safe and non-toxic for biological tissues over prolonged durations.^44^^,^^45^ We find that HG is a suitable replacement medium for water-immersion microscopy that integrates seamlessly into existing optical setups and drastically enhances the stability of intravital imaging.

## 2 Methods

### 2.1 Multiphoton Microscopy and Immersion Media

Data were obtained with a two-photon excited fluorescence (2PEF) microscope optimized for intravital imaging (Bergamo II Series; Thorlabs, Newton, NJ, United States). This system was configured in an upright fashion for live animal preparations. Image translation was facilitated by the movement of the microscope platform with motorized control along cartesian axes, piezo motor control along the optical axis, and an articulating body with up to ±45  deg rotation of the optical axis relative to gravity. Femtosecond laser pulses were generated at 80 MHz from a tunable solid-state ytterbium-doped fiber laser with a usable power band between 680 and 1300 nm and with dispersion compensation (Insight X3; Newport Spectra-Physics, Milpitas, CA, United States). The beam was attenuated by rotating a λ/2 wave-plate relative to a polarizer, then routed onto the microscope platform and scanned by hybrid resonant-galvanometric mirrors, and relay-imaged to the back aperture of a 1.1 NA, 25× water-immersion objective with a working distance of 2 mm and coverslip correction collar to compensate for depth-dependent aberration (CFI75 Apochromat 25XC W; Nikon, Tokyo, Japan). 2PEF signal was collected by the same objective, reflected by a primary dichroic mirror (705 nm long-pass; Thorlabs), split into red and green channels with a secondary dichroic mirror (562 nm long-pass; Semrock, Rochester, NY, United States), spectrally cleaned with additional band-pass filters (Semrock), and relayed to photomultiplier tubes (PMTs; PMT2100 Series; Thorlabs). Microscope control, laser scanning, signal processing, and data acquisition were conducted using ThorImage LS (Thorlabs). Immersion media used in this study included water with a refractive index of 1.333 and surgical-grade HG. HG formulations used for experiments were 1% (w/v) sodium hyaluronate with molecular weights (MWs) of 1.8 or 2.5 million Da and zero-shear viscosities of ∼105  Pa·s3 (Ophthalin, Zeiss, Dublin, CA, United States; Provisc, Alcon, Fort Worth, TX, United States, respectively), with refractive indices of 1.335 to 1.346, depending on the excitation wavelength.^42^^,^^46^^,^^47^

### 2.2 Animals

This study was carried out in strict accordance with National Institutes of Health regulations and the Institutional Animal Care and Use Committee at the University of California, San Francisco, United States. C57BL/6 wildtype mice (Jackson Laboratory, Bar Harbor, ME, United States) were used for deep neurovascular imaging and CX3CR1^eGFP/+^ mice were used for timelapse imaging of retinal microglia expressing enhanced green fluorescent protein (EGFP).^48^^,^^49^

### 2.3 Intravital Imaging

For cortical imaging, C57BL/6 mice were anesthetized with 2% to 4% isoflurane in oxygen, stabilized with a custom stereotax, and implanted with cranial windows as previously described.^9^^,^^33^ The vasculature was labeled with intravenous administration of Texas Red-dextran (50  μL, 1% w/v, MW = 70,000, Sigma-Aldrich, St. Louis, MO, United States) in saline. 2PEF was excited with femtosecond laser pulses centered at 750 nm and spectrally cleaned with a bandpass filter centered at 607 nm. Image stacks were acquired using either water- or HG-immersion. All other imaging parameters were kept identical including laser powers, PMT gain, 900-μm stacks collected in 2-μm steps, and in the same cortical region within the same animal.

For ocular imaging, ${\mathrm{CX}3\mathrm{CR}1}^{\mathrm{eGFP}/+}$ mice were anesthetized with 2% to 4% isoflurane in oxygen, stabilized with a custom stereotax, and the upper eyelid was retracted with a suture (6-0, Silk, Ethicon, Raritan, NJ, United States). A second suture was placed at the superior limbus and used to introduce the globe to expose the superior sclera. For water-immersion imaging, a round 5-mm-diameter coverslip was placed on the eye to create a retention surface and water was added between the coverslip and objective as needed. HG-immersion imaging was performed with and without the coverslip, and the gel was administered once at the beginning of an imaging session. EGFP fluorescence was excited with femtosecond laser pulses centered at 930 nm and spectrally cleaned with a bandpass filter centered at 525 nm. Timelapse image stacks were acquired in 15 s intervals using either water or HG immersion. The image focus was ∼120  μm beneath the ocular surface, and serial stacks of 30-μm depth were collected in 2-μm steps to encompass selected microglial cells in the retina. All optical parameters were kept the same between experiments and across all timepoints including laser powers and PMT gain.

HG was administered into each preparation using a syringe and cannula to avoid air bubbles. After imaging, care was taken to gently clean the objective lens with methanol and lens paper to remove HG before drying. Importantly, once dry, HG formed a persistent residue that was more difficult to clean than typical immersion media. If HG did dry on the objective lens, the tip of the lens was submerged in water for several minutes before being gently wiped once with methanol and lens paper. This process was repeated several times until the HG residue was fully removed. Care was taken to avoid rubbing which might scratch the lens.

### 2.4 Resolution and Signal-to-Background Analyses

To evaluate depth-dependent resolution, fluorescent 0.1  μm beads (TetraSpeck Microspheres; ThermoFisher, Waltham, MA, United States) were suspended in 0.6% agarose gel and 30  μm image stacks were acquired in 0.5  μm steps at depths of 100, 600, and 1100  μm below the surface of the agarose gel, using either water or HG immersion. HG immersion media was 1% sodium hyaluronate with an approximate refractive index of 1.335 at 1040 nm excitation wavelength.^42^ Image stacks were repeated using different correction collar settings on the objective lens to empirically compensate for depth- and media-dependent spherical aberration. Other optical parameters were kept identical including laser powers and excitation centered at 1040 nm, PMT gain, and region of bead suspension. Axial and lateral point-spread-functions (PSFs) of the beads were analyzed using MATLAB code as previously described^50^^–^^52^ where axial and lateral spatial resolutions were defined as the average full-width half-maximum of the axial and lateral PSFs of captured beads, respectively.

Depth- and time-dependent signal-to-background ratios (SBRs) were calculated for intravital imaging and compared between water- and HG-immersion media. For cortical imaging, SBR was determined at varying depths using 40-μm projections centered at 100, 300, 500, 700, and 900  μm beneath the cortical surface. Mean pixel intensity in five regions-of-interest (ROIs) selecting true vascular signal was divided by mean pixel intensity from five ROIs selecting background signal to determine mean SBR at each depth. For retinal timelapse imaging, 30-μm projections were centered on the inner retinal layer to capture microglial cells. The mean pixel intensity from five ROIs selecting EGFP signal in the cell bodies of microglia was divided by the mean pixel intensity from five ROIs selecting background signal to determine mean SBRs at each timepoint. ROIs were standardized to circles of 10 pixels in diameter.

## 3 Results

### 3.1 Depth-Dependent SBR in Mouse Cortical Vasculature

**Fig. 1:**
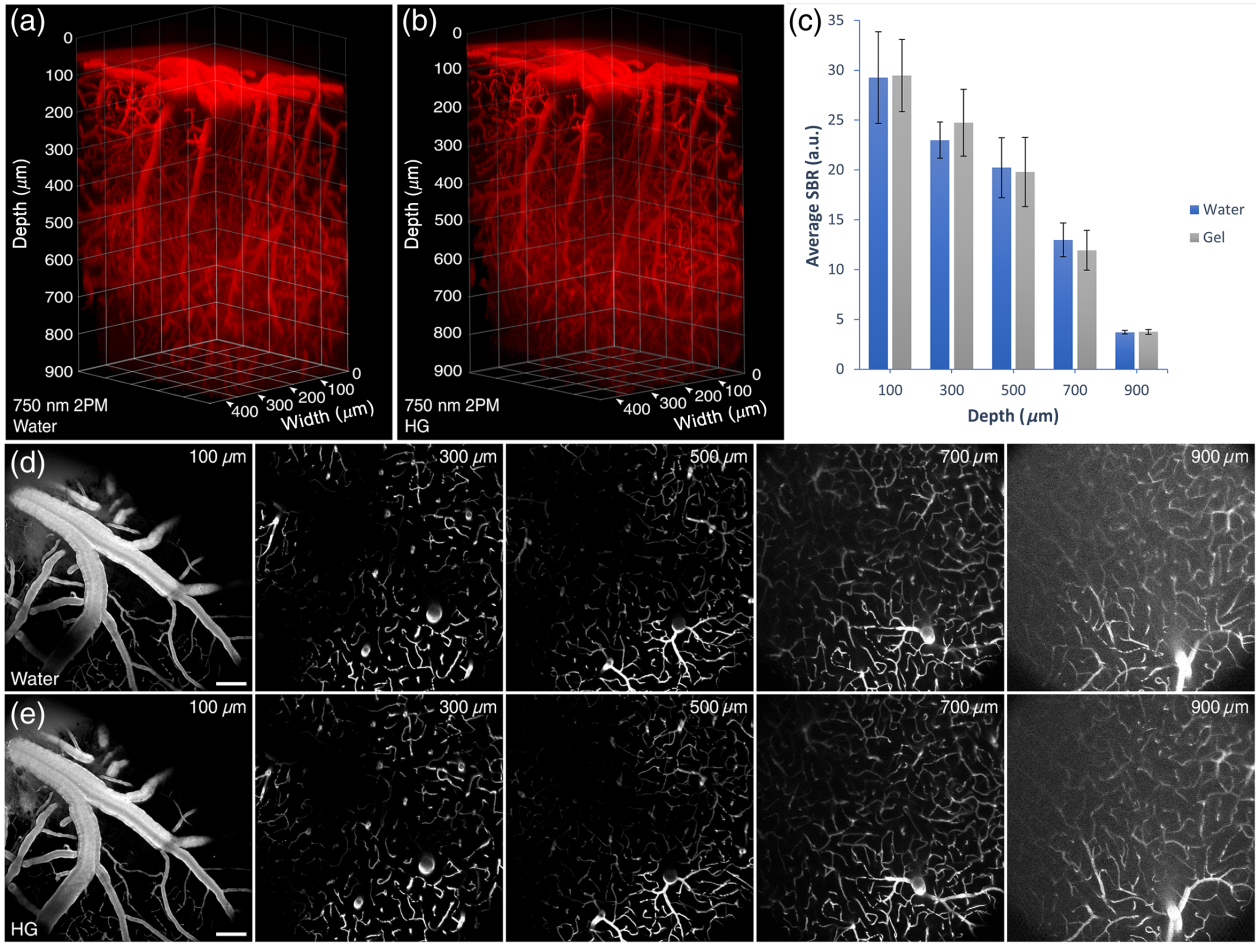
Comparison of depth-dependent 2PEF image quality in mouse cortex with water- or HG-immersion media. (a) 900  μm stack of the mouse cortical vasculature imaged with water immersion. (b) 900  μm stack of the mouse cortical vasculature imaged with HG immersion. (c) Average SBR calculated from optical sections at depths of 100, 300, 500, 700, and 900  μm with water or HG immersion (error bars = ±1 SD). (d) Representative images of the cortex at depths of 100, 300, 500, 700, and 900  μm with water immersion. (e) Representative images of the cortex at depths of 100, 300, 500, 700, and 900  μm using HG immersion. Scale bars=100  μm.

Intravital 2PEF imaging of the mouse cortex revealed equivalent depth-dependent SBR when imaging with water- or HG-immersion media (Fig. 1). The objective’s correction collar was set to 0.17 mm corresponding to the coverslip thickness of the cranial window. SBR decreased with imaging depth without a significant difference between imaging with water or HG, indicating that HG and water are interchangeable as immersion media without degradation of quality when imaging cortical vasculature.

### 3.2 Time-Dependent SBR Ratio in Mouse Retinal Microglia

**Fig. 2:**
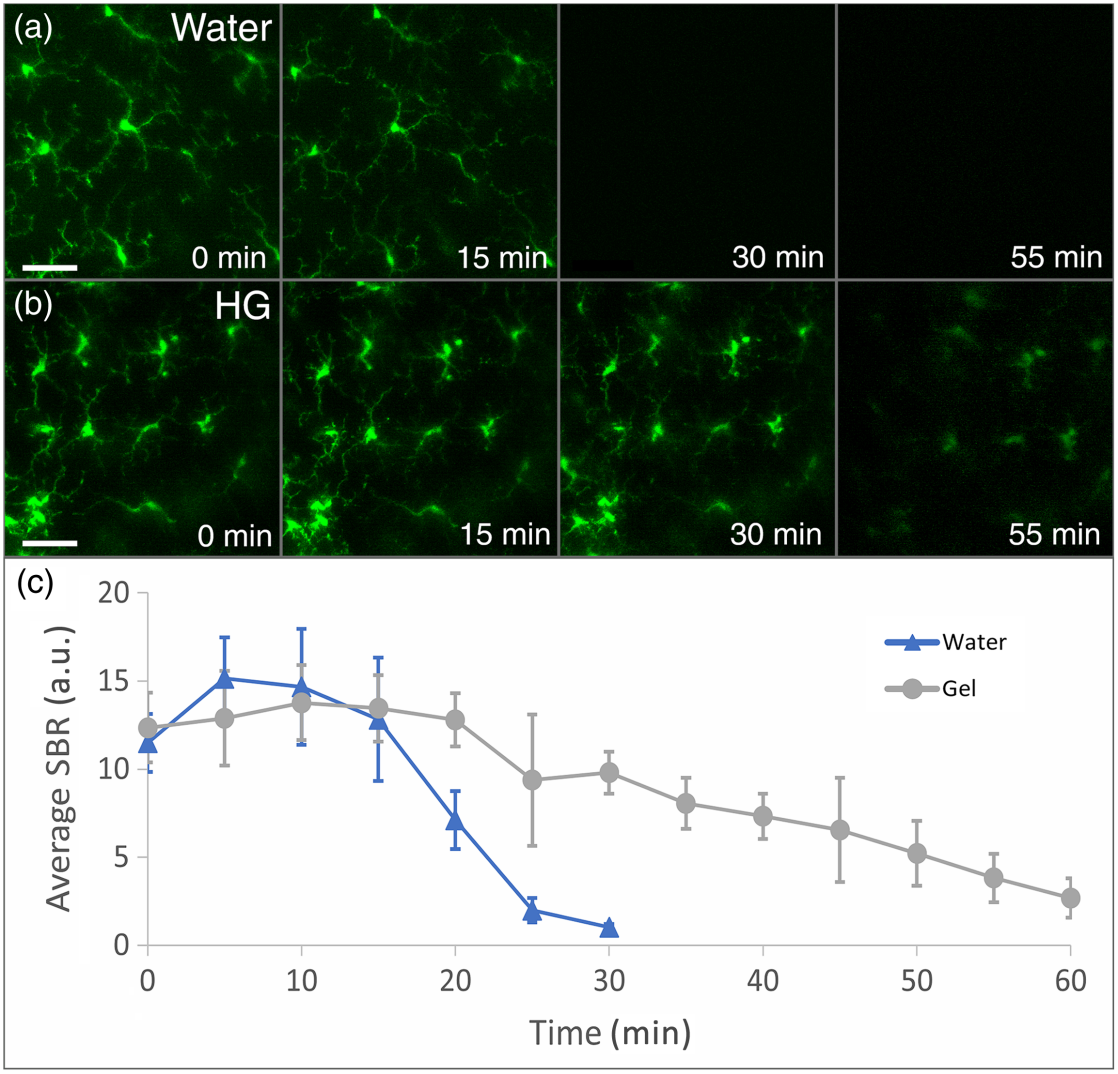
Comparison of time-dependent 2PEF image quality in mouse retina with water- or HG-immersion media. (a) Timelapse imaging of retinal microglia with water immersion, demonstrating a drop-off in SBR to zero within 30 min. (b) Timelapse imaging of retinal microglia with HG immersion, demonstrating gradual drop-off in SBR over the course of 1 h with discernable microglia at the final timepoint. (c) Average SBR calculated from images at serial timepoints using water- or HG-immersion media (error bars = ± 1 SD). Scale bars=40  μm (Video 1, MP4, 7.26 MB [URL: https://doi.org/10.1117/1.NPh.12.S1.S14602.s1]; Video 2, MP4, 14.1 MB [URL: https://doi.org/10.1117/1.NPh.12.S1.S14602.s2]).

Timelapse imaging with water demonstrated a complete drop-off in SBR within 30 min [Fig. 2(a) and 2(c), Video 1], whereas timelapse imaging with HG had a gradual drop-off in SBR over the course of 1 h where microglia and their subcellular processes were still visible at the final timepoint [Figs. 2(b)–2(c), Video 2]. Immersion media was not replenished in this experiment, and the decrease in signal was likely attributable to wicking and evaporative loss of immersion fluid. SBR with water immersion was slightly higher than HG immersion at the beginning of timelapse imaging. Notably, the correction collar of the objective lens was set to 0.17 mm per the manufacturer’s specifications, corresponding to the glass coverslip thickness when imaging under water immersion. We therefore sought to quantitatively evaluate image quality with either water or HG immersion at various imaging depths and to determine if this could be optimized by adjusting the correction collar on the microscope objective.

### 3.3 Depth-Dependent Resolution

**Fig. 3:**
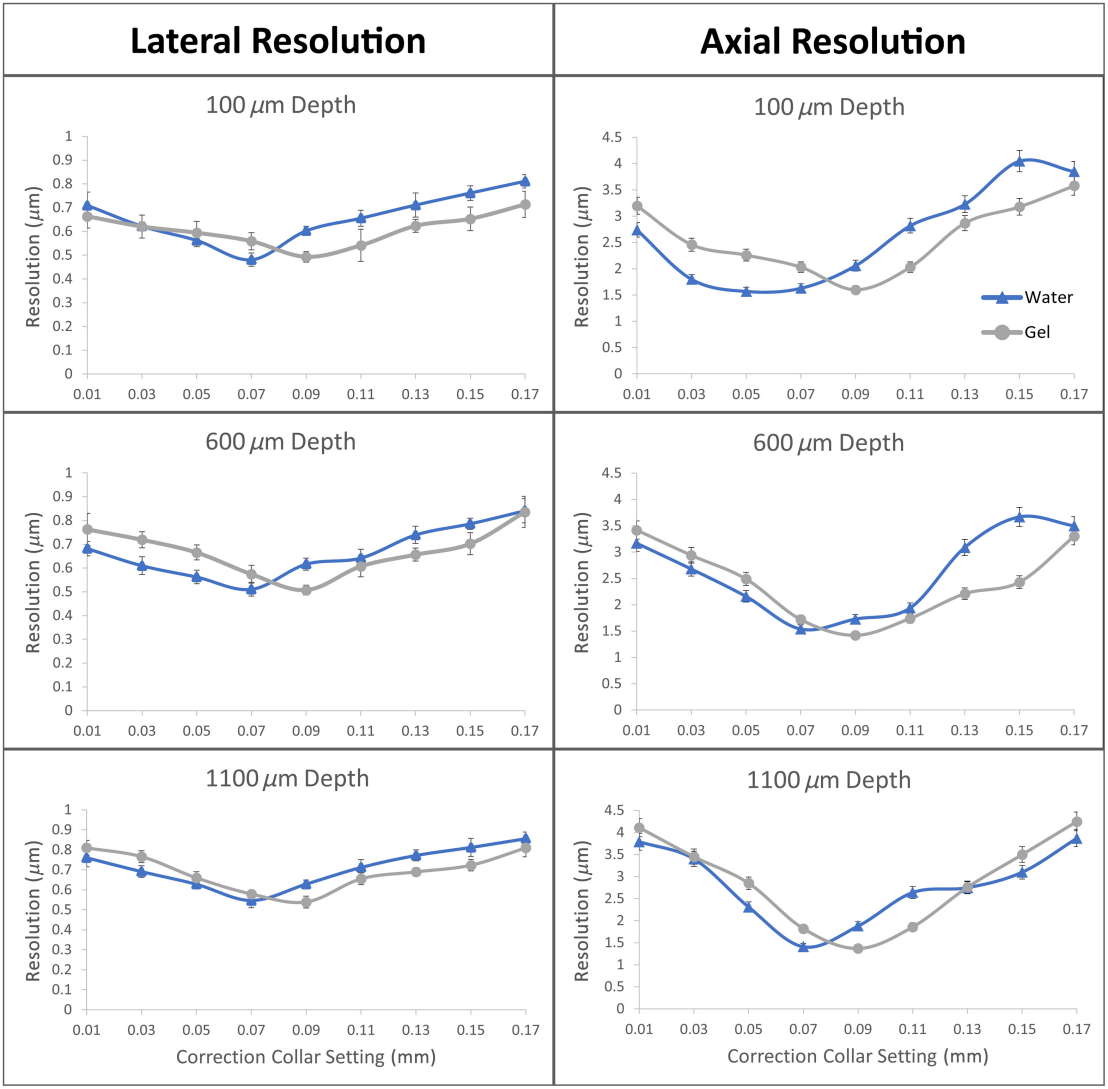
Comparison of lateral and axial resolution at varying depths and objective correction collar settings. PSFs of 0.1  μm beads were analyzed to obtain lateral and axial resolutions at depths of 100, 600, and 1100  μm and with different correction collar settings. Peak resolutions were equivalent for HG and water at every depth when optimized with the correction collar. Correction collar settings are reported in units of mm corresponding to the manufacturer’s intended use with varying cover glass thicknesses (error bars = ± 1 SD).

Peak lateral and axial resolutions were equivalent using water and HG at all depths with optimal correction collar settings. Correction collar settings are reported in units of mm corresponding to the manufacturer’s intended use with varying cover glass thicknesses. For water immersion, peak resolutions were achieved with a correction collar setting of 0.07 mm. Lateral resolutions in water peaked at 0.48, 0.51, and 0.55  μm at depths of 100, 600, and 1100  μm, respectively. Axial resolutions in water peaked at 1.57, 1.54, and 1.41  μm, at depths of 100, 600, and 1100  μm, respectively (Fig. 3). For HG immersion, peak resolutions were achieved with a correction collar setting of 0.09 mm. Lateral resolutions in HG peaked at 0.49, 0.51, and 0.54  μm at depths of 100, 600, and 1100  μm, respectively. Axial resolutions in HG peaked at 1.6, 1.42, and 1.37  μm, at depths of 100, 600, and 1100  μm, respectively (Fig. 3). These results indicate that the replacement of water with HG as an immersion medium enables equivalent imaging resolution when optimized with a small adjustment in correction collar settings.

### 3.4 Stability of Water and Hyaluronan Gel

**Fig. 4:**
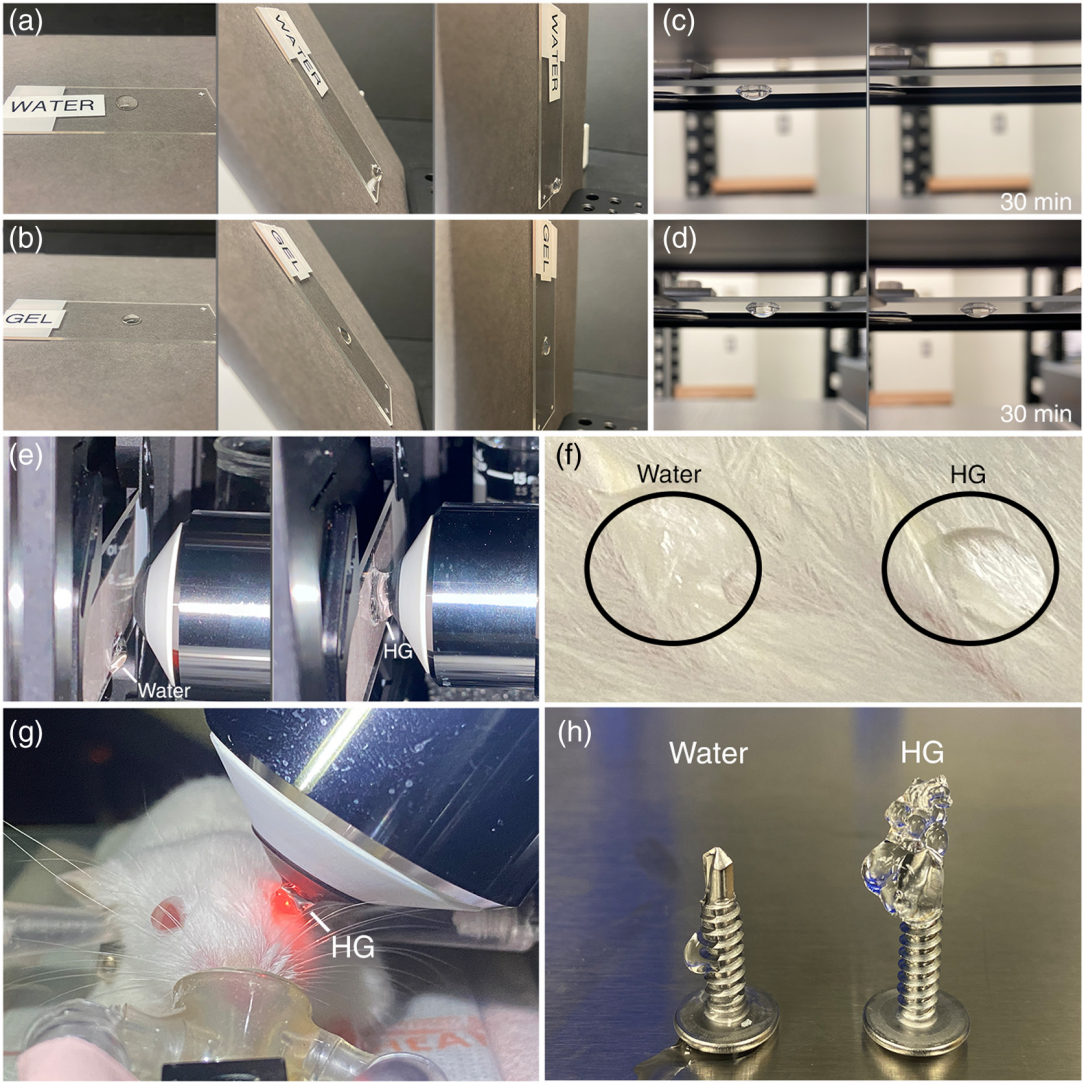
Comparative stability of HG and water. Adhesion of a droplet of (a) water and (b) HG to a glass slide at 0, 60, and 90 deg from 3 mm, respectively. Adhesion of (c) water and (d) HG to a glass slide inverted to gravity for up to 30 min. Adhesion of (e) water and HG between a glass slide and water-immersion objective separated by 3 mm. (f) Wicking of water and HG on mouse fur, demonstrating resistance of HG to wicking. (g) Example of HG usage with an intravital imaging preparation unamenable to water. (h) Comparison of water and HG cohesion and stability on geometry with a small surface area.

HG demonstrated enhanced stability in configurations that are traditionally challenging with water. When applied on glass slides, HG remained in its initial position without moving even when oriented 90 deg from horizontal, whereas water consistently drifted to the edge of the slides at oblique angles [Figs. 4(a) and 4(b)] (Fig. S1 in the Supplementary Material) In an inverted orientation, HG showed no displacement and only a slight reduction in volume due to evaporation after 30 min, whereas water dripped or evaporated quickly from the slide [Figs. 4(c) and 4(d)] (Fig. S2 in the Supplementary Material). HG was easily maintained between a glass slide and a water-immersion objective separated by a distance of 3 mm and oriented 90 deg from horizontal, a condition not possible with water [Fig. 4(e)] (Fig. S3 in the Supplementary Material). On mouse fur, HG was stable and retained its cohesive structure, whereas water was prone to wicking and absorption [Fig. 4(f)]. Figure 4(g) presents an example of HG application for ocular imaging at an oblique angle with limited surface area and surrounding fur, a preparation unamenable to water. Figure 4(h) illustrates the high stability and cohesion of HG on the tip of a metal screw with limited surface area.

## 4 Discussion

The use of viscocohesive HG as an immersion medium for intravital MPM opens the doors to new imaging assays and avenues of research. This approach leverages the development of viscosurgical devices, or “viscoelastics,” for intraocular surgery. Viscosurgical devices come in a range of formulations which are typically mixtures of water and sodium hyaluronate with concentrations between 0.8% to 3% and MWs from 800,000 to 5,000,000 Daltons and can occasionally include chondroitin sulfate.^44^^,^^45^^,^^53^ The composition of HG determines its viscous, dispersive, and cohesive properties which are designed to preferentially immobilize and protect different tissues of the eye while maintaining refractive indices closely aligned with water, usually ranging from 1.335 to 1.346.^41^^–^^43^^,^^46^^,^^53^ Thus, HGs can be implemented easily with existing water-immersion setups without altering optics or hardware, are biologically safe, and offer exceptionally high viscosities and cohesiveness, which enable challenging or previously impossible imaging configurations.

There are several limitations and technical considerations when implementing HGs as replacement media for water-immersion imaging. First, it is important to avoid bubbles when delivering HG between the specimen and objective as these will remain trapped and can degrade image quality. If bubbles are present, HG can be removed and reapplied without bubbles, or additional HG can be applied to displace bubbles from the optical path. This also makes HGs impractical for assays requiring continuous bubbling of oxygen or nutrients in media as with certain tissue cultures.^54^ We find that delivering HG slowly with a 27-gauge syringe or 27-gauge blunt-tip cannula (∼210  μm inner diameter) is an excellent approach to avoid bubbles. Second, the water content of HG slowly evaporates causing the gel to become more concentrated, which over extended periods may influence image quality and shift the effective focal plane. This change can be mitigated by replenishing the HG or re-wetting the existing gel periodically with a drop of water. In our experience, HG with periodic wetting every ∼20  min lasts hours without apparent changes in image position and quality. Third, cleaning optics is important and different when using HG as an immersion medium. Although chemically inert, HG can dry into a persistent residue. Immersion lenses should be cleaned immediately after experiments to avoid drying of HG on the optic surface. However, if drying does occur, the HG residue should be rehydrated to facilitate non-abrasive removal by submerging the tip of the optic in water for ∼5  min, followed by careful cleaning with lens paper and methanol. This hydration process can be repeated if there is still HG residue on the optic. Importantly, rubbing the dry optic should be avoided to prevent potential scratching. Fourth, clinical-grade HGs are manufactured for human surgery and can be costly. Ingredients such as sodium hyaluronate are relatively inexpensive and HG could be made in the laboratory to save on cost and optimize formulations to specific experimental needs. In principle, HGs can also be designed with a much higher index of refraction than water, which if paired with custom optics, might further enhance image resolution, working depths, and gel cohesiveness for intravital imaging preparations.

The implementation of HG with intravital MPM paves the way for new and previously infeasible imaging experiments. While our study highlights the potential of HG in multiphoton imaging, its versatility may extend broadly to other water-immersion microscopy techniques. As a viscous interface, HG is also applicable to ultrasonography, which could facilitate multimodal imaging in conjunction with optical methods. Notably, the use of HG with microscopy has minimal cost or complexity, is readily accessible to the broader research community, and has great potential for facilitating novel imaging experiments and discovery in biomedical research.

## Supplementary Material







## Data Availability

The data presented in this paper will be uploaded to an open access repository.
