# Supplementary material for: Viscocohesive hyaluronan gel enhances stability of intravital multiphoton imaging with subcellular resolution
Source: Neurophotonics. 2024 Nov 22;12(Suppl 1):S14602. doi: 10.1117/1.NPh.12.S1.S14602 (PMC11582905; doi:10.1117/1.NPh.12.S1.S14602)
Supplement: Supplementary file 1 [file NPh_012_S14602_SD001.pdf]

Supplementary Material

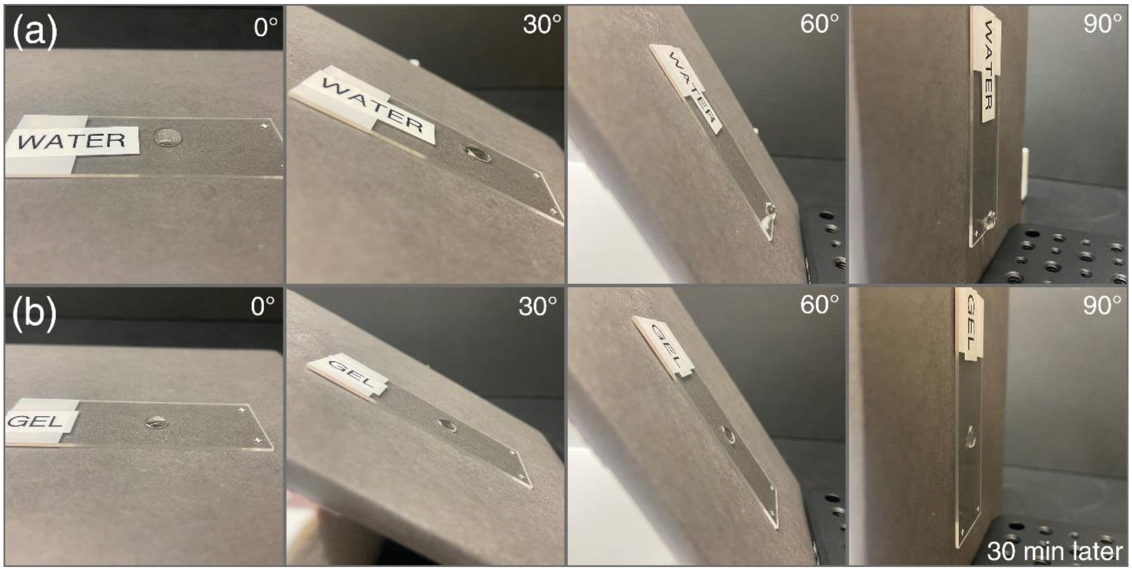

Fig. S1 Adhesion at varying angles. (a) Adhesion of small droplet of water at angles 0, 30, 60, and 90 degrees. At 30 degrees, the water droplet begins to flatten and drift from its original position. At 90 degrees, the droplet has traveled to the edge of the glass slide. (b) Adhesion of a small droplet of HG to a glass slide at angles 0, 30, 60, and 90 degrees, demonstrating no movement and minimal change in shape, even after 30 minutes of being left in a vertical position.

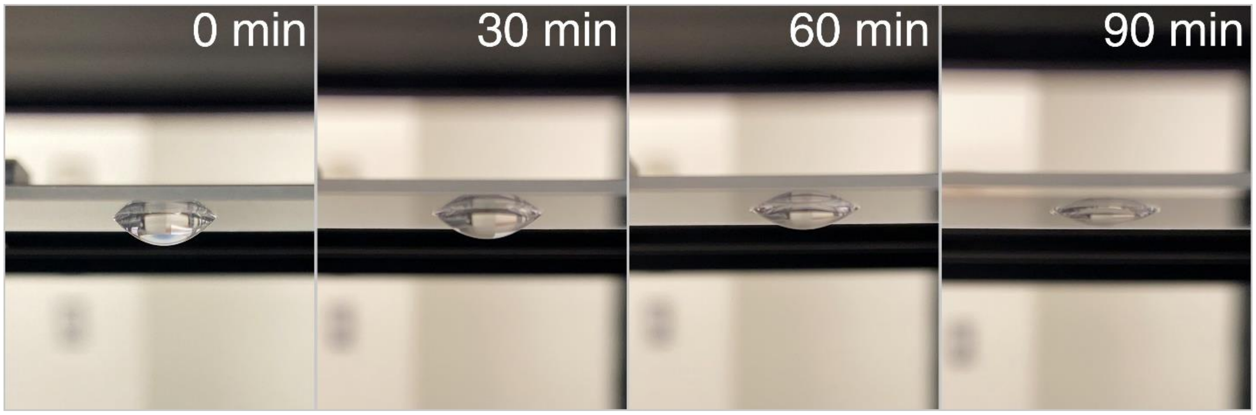

Fig. S2 Inverted stability and evaporation. To demonstrate the resistance of HG to evaporation and its overall stability, we show the gel's presence over 90 minutes on a glass microscope slide in an inverted position. HG is still substantially present after 60 minutes, but gradually loses volume over time due to evaporation.

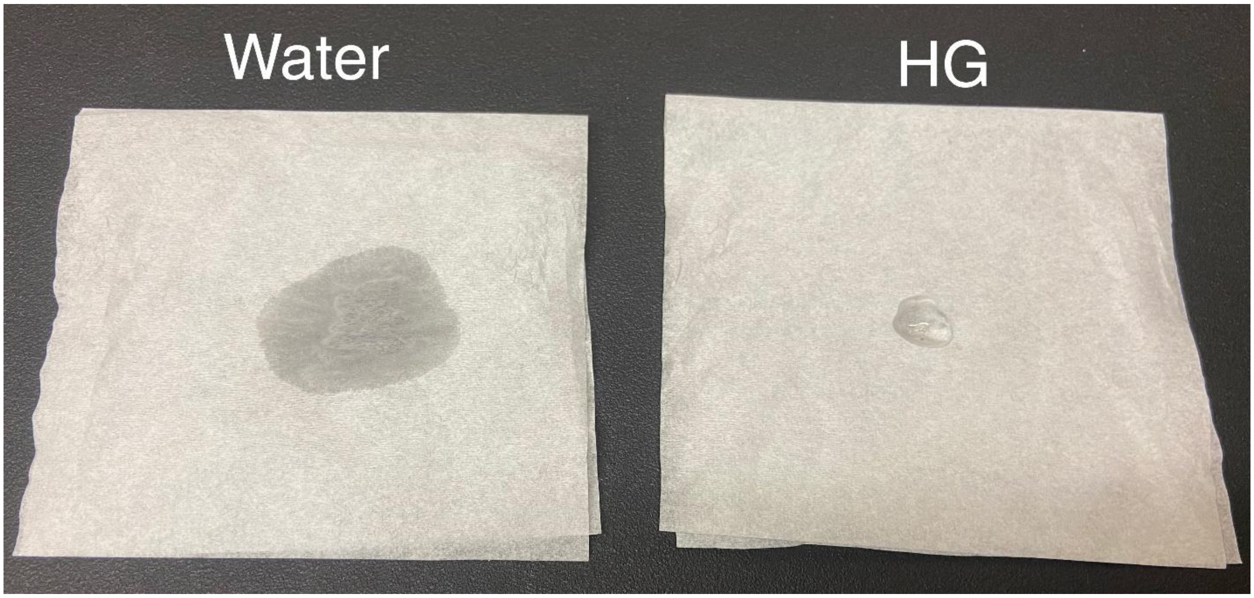

Fig. S3 Wicking Resistance. To demonstrate the resistance of HG to wicking, we simply show the placement of a water droplet and a gel droplet on absorptive tissues. Photos were taken immediately after placement of both droplets.
